# Anaemia Profile and Inflammation Markers in Stunted Children Under Two Years in Indonesia

**DOI:** 10.3390/children11111315

**Published:** 2024-10-29

**Authors:** Luhung Budiailmiawan, Aryati Aryati, Nursin Abd. Kadir, Laily Indrayanti Yusuf, Lia Gardenia Partakusuma, Louisa Markus, Leni Lismayanti

**Affiliations:** 1 Palabuhanratu Hospital, Sukabumi 43364, West Java, Indonesia; luhungbudiailmiawan@yahoo.co.id; 2 Departement of Clinical Pathology, Faculty of Medicine, Universitas Airlangga, Surabaya 60286, East Java, Indonesia; 3 Institute of Tropical Diseases, Universitas Airlangga, Surabaya 60115, East Java, Indonesia; 4 Departement of Clinical Pathology, Faculty of Medicine, Universitas Hasanudin, Makassar 90245, South Sulawesi, Indonesia; nursinak@gmail.com; 5 General Hospital of West Nusa Tenggara Province, Mataram 84371, West Nusa Tenggara, Indonesia; lely_indrayanti@yahoo.com; 6 Post Graduate Programme, Faculty of Medicine, YARSI University, Jakarta 10510, DKI Jakarta, Indonesia; lia.gardenia@yarsi.ac.id; 7 Regional General Hospital Cengkareng, Jakarta 11730, DKI Jakarta, Indonesia; alamatkuyangbaru@hotmail.com; 8 Departement of Clinical Pathology, Faculty of Medicine, Padjadjaran University, Dr. Hasan Sadikin Central General Hospital, Bandung 40161, West Java, Indonesia; leni.lismayanti@unpad.ac.id

**Keywords:** stunting, anaemia profile, inflammatory markers

## Abstract

**Background:** Stunting is a common issue affecting children who suffer from chronic malnutrition in Indonesia. The Indonesian government has introduced supplementary food programs for stunted children, but the results have been less satisfactory. This may be due to the presence of anaemia and comorbid diseases. Haematology tests and inflammation markers are necessary to identify these conditions. This study aimed to examine the anaemia profiles and inflammation markers in stunted children under two years old. **Methods**: A cross-sectional descriptive design with cluster samples and consecutive analysis was used. The study was conducted between December 2023 and March 2024 at the West Nusa Tenggara Hospital and Palabuhanratu Sukabumi Hospital laboratories. Samples were obtained from various Public Health Centres in Sukabumi, West Java, North Maluku, and West Nusa Tenggara. Data collection comprised interviews, measurements, and the assessment of haematology, biochemical, and inflammatory markers. Statistical analysis was conducted using SPSS version 20, which includes descriptive analysis, correlation, comparison, and chi-square tests. **Results**: Two hundred and ten stunted children were identified with various anaemias and comorbidities. These anaemias included suspected thalassemia (38.1%), iron deficiency (18.1%), and anaemia of chronic diseases (13.3%). Based on the inflammatory markers obtained, TB was suspected (21.4%), inflammatory bowel disease (18.1%) was suspected, and allergic proctocolitis was suspected (31.9%). **Conclusions**: Analysis of the anaemia profiles and inflammatory markers revealed various types of anaemia and suspected comorbidities in stunted children. It is recommended that anaemia profiles and inflammation markers be assessed at the primary healthcare level.

## 1. Introduction

Malnutrition is a significant global problem among infants and toddlers. The World Health Organization (WHO) estimates that the stunting rate in 2022 will be around 22.3% or 148 million children worldwide million children. Overweight also impacted an estimated 5.6% or 37 million children, while wasting endangered the lives of approximately 6.8%, or 45 million children [1]. The global prevalence of stunting is categorised as high, ranging from 20% to 30%. This percentage may increase significantly due to constraints in accessing essential food and nutrients during the COVID-19 pandemic. According to the 2023 Global Hunger Index (GHI), Indonesia is ranked 77th out of 125 countries, falling within the middle to lower middle range. The prevalence of malnutrition and wasting in toddlers is one of the indicators of GHI [2].

Based on the 2022 Survei Status Gizi Indonesia (SSGI), stunted cases decreased in 2021 from 24.4% to 21.6%. The prevalence of stunted toddlers in West Nusa Tenggara, North Maluku, and West Java was estimated at 32.7%, 26.1%, and 20.2%, respectively. Sukabumi Regency had the second-highest number of stunted toddlers in West Java after Sumedang at 27.5%, while Central West Nusa Tenggara Regency had the highest cases at 37%. The instances in Ternate and Tidore, North Maluku, were estimated at 19.1% and 17.7%, respectively [3].

The definition of stunting based on the WHO is a condition characterised by short stature compared to children of the same age. A height-for-age z-score (Z-score/HAZ) greater than two standard deviations of the Median Child Growth Standard indicates stunting [4]. Based on Riset Kesehatan Dasar (Riskesdas), there was a decrease in the stunting rate from 37.6% in 2013 to 30.8% in 2018. Data from the Survei Status Gizi Indonesia (SSGI) further showed a decrease from 24.4% in 2021 to 21.6% in 2022 [3].

Stunting begins in utero and continues postnatally for at least the first two years of life. The average z-score for body length for age in newborns in developing countries is around −0.5, then continues to decline postnatally until it reaches 2.0 at 18–24 months of age [5]. Adolescent growth increases between two and mid-childhood, and linear growth may be delayed. However, the impact during this period on other components of stunted growth syndrome, such as cognitive and immune function, is impaired. The period from conception to a child’s second birthday is the most critical window of opportunity for interventions in stunted children [6].

Stunting and anaemia are public health problems that are most frequently found in developing countries [7]. Anaemia is when the body’s physiological needs are unmet due to insufficient red blood cells and decreased oxygen-carrying capacity. Generally, anaemia in stunted children is caused by iron deficiency [8]. Children under the age of five are particularly vulnerable to anaemia and stunting; this creates a heavy burden for individuals and the country. This can cause long-term impacts, including poor nutrition, which hinders children’s development and potentially affects adult life. For example, adults who were stunted as children may experience declining productivity, low education, and poor knowledge, ultimately contributing to an 8% decline in Indonesia’s economic growth [9].

Haematology, serum iron, and ferritin laboratory tests are crucial for screening and differentiating anaemia in stunted children. The Mentzer index (MI) test (MCV/RBC < 13) and the Shine and LaI index ([MCV × MCV × MCH/100] < 1530) are useful for early β-thalassemia carrier detection [10]. The Mentzer index demonstrated 98.7% sensitivity and 82.3% specificity for early thalassemia screening [11]. The Shine and Lal Index (SLI) helps in the initial screening of β-thalassemia carriers as well as confirming mutations in CD-26 (c.79 G > A) and in IVS1nt5 (c.92 + 5 G > C), which are both common mutations in Bandung, Indonesian [10].

The neutrophil-to-lymphocyte Ratio (NLR) and platelet-to-lymphocyte Ratio (PLR) are considered inflammation markers. As a key factor, inflammation has been previously associated with the malnutrition–inflammation–atherosclerosis cascade and calcification syndrome. Elevated platelets, neutrophils, and decreased lymphocyte counts represent some haematological abnormalities in severe acute malnutrition children [12]. The NLR and PLR serve as straightforward and cost-effective indicators of inflammation, easily derived from differential WBC counts. Nevertheless, neither can accurately depict the inflammation process in stunted children. Body mass index (BMI) status has also been shown to significantly influence blood cell counts, specifically the number of lymphocytes, neutrophils, and platelets. Furthermore, Domnicu et al. reported an NLR >1.43 showing bacterial infections in malnutrition toddlers, with 85% sensitivity and 69% specificity [13].

The platelet-to-lymphocyte ratio (PLR) predicts the clinical outcome of individuals with systemic inflammation better than other counts, such as platelets or lymphocytes. Hypercortisolaemia caused by any degree of stress with a secondary release of platelets into the bloodstream and transient lymphopenia predisposes to increased levels of PLR in a large number of proinflammatory and prothrombotic diseases. The non-specific pathogenetic cascade, including increased PLR, can be neutralised by intensive platelet destruction or consumption at the site of inflammation and thrombosis. This requires checking all blood cell counts as well as various inflammatory and serological markers. Gysi et al. reported that an increase in PLR value >151 showed a rise in malnutrition in hospitalised children, with a sensitivity of 51.6% and a specificity of 67.3% [12]. Furthermore, Zahmatkesh, et al. focused on the ratio of platelets/lymphocytes and neutrophils/lymphocytes to diagnose and assess the severity of inflammatory bowel diseases (IBD) in children. The results showed that an NLR value > 2.04 had a sensitivity of 82.1% and a specificity of 82.9%, while a PLR > 103 had values of 67.9% and 71.4%, respectively, for detecting IBD [14].

Mean platelet volume (MPV) increases in various chronic diseases, such as malignancy and inflammation. The platelet surface expresses IgE receptors; hence, some allergic diseases cause an increase in MPV values. Allergens activate platelets, releasing mediators such as platelet factor 4, β-thromboglobulin, RANTES, and thromboxane. Aratisma Makalesi’s study showed a significant increase in MPV in children with allergic proctocolitis with an average value of 9.07 ± 0.78 (*p* < 0.001) [15].

The host–pathogen TB complex interaction has given rise to a variety of innate and adaptive immune mechanisms. Early studies on rabbits and children in the 1920s showed an association of TB disease with increased monocyte and decreased lymphocyte numbers, resulting in an increased monocyte-to-lymphocyte ratio (MLR) [16]. Kissling et al. reported an MLR value of >0.44 in children who tend to have tuberculosis (TB) with 92% sensitivity and 32% [17]. Another study conducted by Cursi et al. stated that the value of the neutrophil-to-lymphocyte + monocyte ratio (NMLR) > 1.2 in children showed a tendency toward TB infection with 63% sensitivity and 76% specificity (AUC: 0.72). Meanwhile, MLR > 0.2 in children showed a tendency towards TB infection with a sensitivity of 56% and a specificity of 82% (AUC: 0.71) [18].

The Indonesian government is making efforts to tackle the stunting problem with various programs but has not produced optimal results. For example, additional food programs have been provided to improve the nutrition of children under two years with stunting. However, the weight gain is not significant, which may be related to underlying or accompanying diseases. The Indonesian Association of Clinical Pathology Specialists (PDS PatKLin) felt the need to perform laboratory examinations for stunted children to detect these diseases. To detect these diseases, haematological examination and inflammatory markers are needed to detect underlying or accompanying diseases in stunted toddlers. These inflammatory markers must be feasible in the first level of healthcare facilities and become the basis for treating children under two years with stunting. Therefore, this study aimed to examine the anaemia profiles and inflammatory markers in stunted children under two years old.

## 2. Materials and Methods

### 2.1. Study Design and Setting

This study employs a cross-sectional descriptive design, utilising cluster and consecutive sampling methods for data collection. Based on the sample size formula for Categorical Descriptive, the minimum sample size for this study is 200 samples. The sample was divided into three regions, with a minimum of 60 samples for each area (cluster sample). The inclusion criteria for the sample are stunted children aged from one month to twenty-four months (two years). It was conducted at the Regional General Hospital Laboratory (RSUD) of West Nusa Tenggara Province (NTB) and Palabuhanratu Hospital, Sukabumi Regency. Samples were obtained from several public health centres (Puskesmas) in the public health office (Dinas Kesehatan) in the Sukabumi Regency area, North Maluku, and Teruwai Village, Central West Nusa Tenggara Regency, West Nusa Tenggara from December 2023 to March 2024.

Data were collected using the interview method with questionnaire tools, including information on child characteristics, parent, family income, immunisation status, and parent and child disease history. Moreover, data were collected through measurements such as stunting, the mother’s nutritional status, haemoglobin levels, serum iron levels, ferritin levels, NLR, PLR, and MPV (File S1).

### 2.2. Study Participants

**Figure 1:**
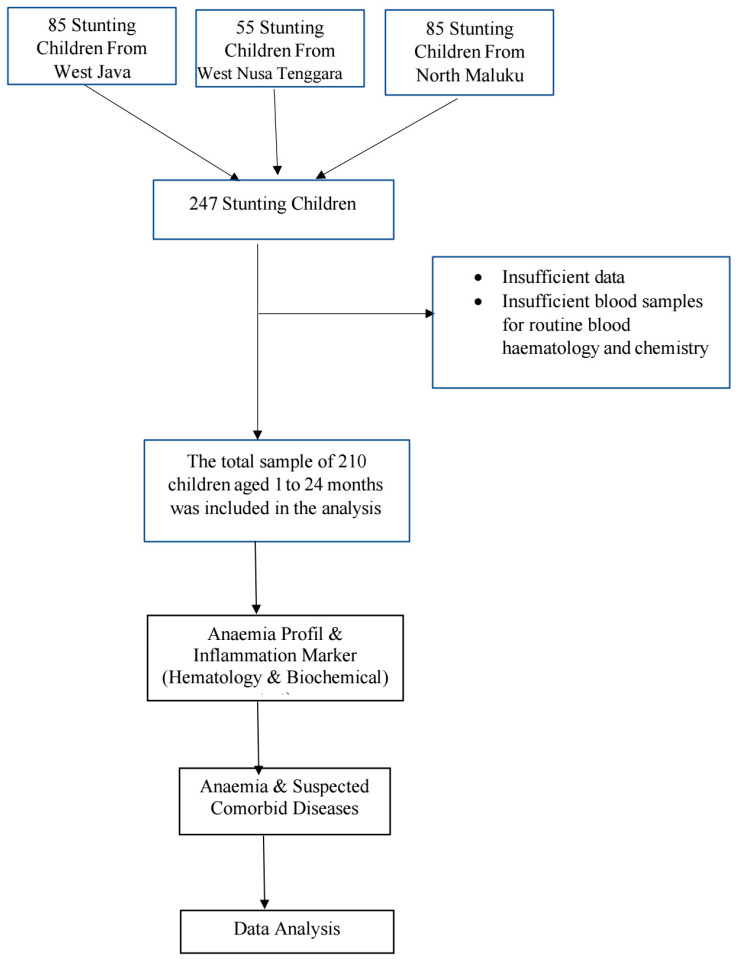
The research flowchart.

The participants were confirmed to be stunted children aged from one month to two years old. The sample size was determined using the categorical descriptive formula. According to the calculation, the sample size was 247 and was selected through simple random sampling. A total of 37 children were excluded due to insufficient data and blood samples for routine blood and chemistry tests. A research flow chart detailing the inclusion and exclusion of research participants is shown in Figure 1.

Finally, the analysis included a total sample of 210 children aged one to twenty-four months. Syamsudin S.H. Hospital Research Ethics Committee approved the study protocol with reference number 17/KEP-RS/RSUD/XII/2023 (11 December 2023).

### 2.3. Research Questioner Instrument

Mothers/caregivers must sign a written informed consent before participating in this study. Interviews were conducted by health workers trained in previous research questionnaire instruments, using language understood by the local community, and the research instrument used closed questions to minimise bias. Socioeconomic and childhood-related data were collected using standard questionnaires at the beginning of the face-to-face interview. Key elements include (1) child information, namely, gender, age, gestational age, breastfeeding history, age at introduction of complementary foods, immunisation status, and history of child illnesses; and (2) family-specific information, such as socioeconomic characteristics of parents and family, including mother’s education level, mother’s age at marriage, mother’s occupation, mother’s anaemia status, father’s occupation, family income, and family history of illness.

### 2.4. Anthropometric Measurement

Anthropometric measurements for stunted children include the weight and height of the child and parents. Trained health workers measure physical health, measuring each weight to the nearest tenth of a kilogram or one decimal place using the Endo Anthropometric Kit 10 (Endo International plc, Dublin, Ireland). The height of stunted children was measured to the nearest millimetre using a Seca plastic height board (model 213). The nutritional status of stunted children based on height for age (HAZ) is assessed using WHO child growth standards (graphic assessment of body length/height for age in the health card book) and categorised as stunting (HAZ < −2 standard deviations (SD)) or normal (HAZ ≥ −2 SD) [19]. Parental height is categorised as short and normal, including father (short stature [<160 cm] and normal [≥160 cm]) and mother (short stature [<150 cm] and normal [≥150 cm]). To determine the nutritional status of parents based on the Body Mass Index (BMI), which is calculated based on body weight in kilograms divided by height in meters squared and classified according to WHO Asia-Pacific criteria including underweight [<18.5 kg/m^2^], average [18.5–22.9 kg/m^2^], and overweight/obese [≥23 kg/m^2^] [20].

### 2.5. Biochemical and Haematology Measurement

The biochemical assessment was used to evaluate complete haematology (routine haematology and differential cell blood count), serum iron, and ferritin levels. The biochemical evaluation of haematology and iron parameters plays a vital role in diagnosing and managing various haematological conditions, especially those associated with iron metabolism. Nonetheless, iron metabolism cannot account for all the causes of stunted children.

A venous blood sample of 3 cc was collected, including 1 cc placed in an EDTA tube and 2 cc in a chemical tube with a gel separator. The samples were assigned a registration number based on the name and registration number on the case report form, then stored in a cool box and dispatched to the laboratories at Palabuhanratu Regional and West Nusa Tenggara Provincial Hospital.

A haematological examination was conducted using a Mindray BC5380 haematology analyser (Shenzhen Mindray Bio-Medical Electronics Co., Ltd., Shenzhen, China). A serum iron examination was performed on a Mindray chemistry analyser BC-240 (Shenzhen Mindray Bio-Medical Electronics Co., Ltd., Shenzhen, China), and a ferritin examination was conducted using a Mindray Enzyme Chemiluminescent Immunoassay (ECLIA) CL-900i instrument (Shenzhen Mindray Bio-Medical Electronics Co., Ltd., Shenzhen, China). The differential cell blood count from BC 5380 measured inflammatory markers. The Neutrophil-to-Lymphocyte Ratio (NLR) examination was conducted manually by dividing the number of absolute neutrophils (/mm^3^) by the absolute number of lymphocytes (/mm^3^). An NLR > 1.43 shows the possibility of a bacterial infection [13]. The Platelet-to-Lymphocyte Ratio (PLR) examination was conducted manually by dividing the absolute platelet count (/mm^3^) by the absolute lymphocyte count (/mm^3^). A PLR > 103 shows the possibility of IBD [12]. The MLR test was performed manually by dividing the number of absolute monocytes (/mm^3^) by the absolute number of lymphocytes (/mm^3^). An MLR greater than 0.44 in children shows a tendency towards Mycobacterium tuberculosis infection [16]. The NMLR test was conducted manually by dividing the number of absolute neutrophils (/mm^3^) by the absolute number of lymphocytes (/mm^3^) plus the number of absolute monocytes (/mm^3^). An NMLR greater than 1.2 in children shows a tendency towards TB disease [18]. The MPV was obtained from measurements using a haematology analyser. A value > 9.07 in children suggests the potential presence of food protein-induced allergic proctocolitis [15].

### 2.6. Differential Diagnosis of Anaemia Microcytosis

Anaemia and stunting have risk factors that influence each other due to micronutrient deficiencies, including protein and micronutrient deficiencies, specifically iron deficiencies, as well as other substances such as folate, riboflavin, vitamin B12, and vitamin A [21]. Certain body conditions affect anaemia, specifically acute and chronic inflammation. The definition of anaemia in children below 59 months (toddlers) is haemoglobin below 11 mg/dL [8]. The anaemia seen in toddlers is primarily due to iron deficiency (hypochromic microcytosis), attributed to increased demand and use of iron reserves at birth, followed by insufficient intake. The conclusion lacks sufficient justification, as thalassemia is also prevalent in Indonesia. Laboratories markers to differentiate microcytic from normocytic anaemia include The study on paediatric patients with multiple food allergies found that abnormal production of TGF-β in the small intestine is a critical immunological issue using the mean corpuscular volume (MCV < 74 fL) and mean corpuscular haemoglobin (MCH < 27 pg) as a benchmark, which shows microcytic anaemia. Ferritin shows the storage of iron in the tissue, and patients with iron deficiency tend to have low serum iron levels (<22 µg/dL) along with low ferritin levels (<6 ng/mL) [22]. On the other hand, patients with chronic diseases or infections typically show low or normal iron levels (22–136 µg/dL) and elevated ferritin levels (>24 ng/mL). Thalassemia patients usually have normal to elevated iron levels with high ferritin levels [23]. The laboratory tests outlined in Table 1 are essential for accurately diagnosing anaemia microcytosis.

**Figure 2:**
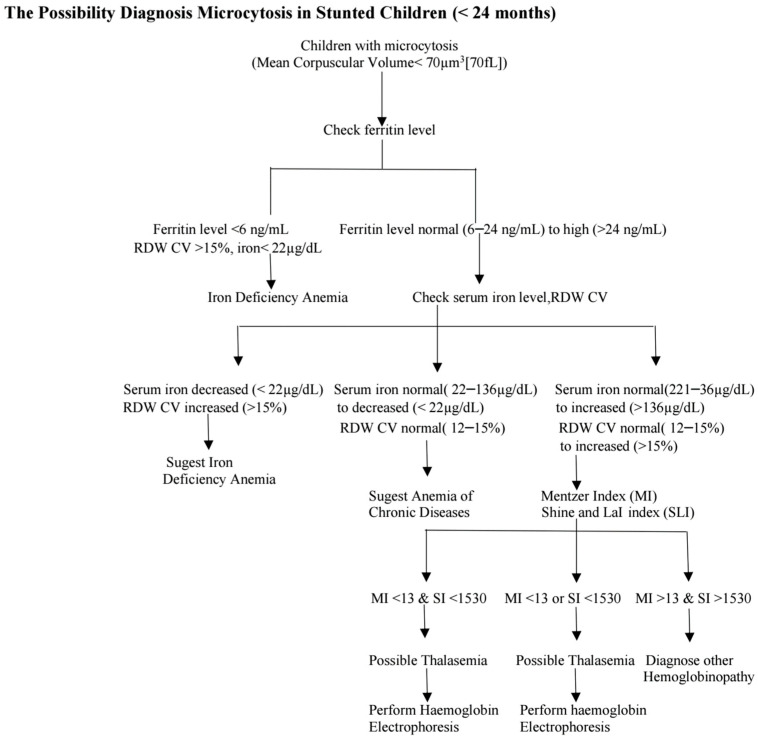
Suggested Algorithm for Diagnosing the Cause of Microcytosis in Stunted Children (<24 Months).

Mentzer Index (MI) (MCV/RBC < 13) and Shine and LaI index ([MCV × MCV × MCH/100] < 1530) are employed in the screening for thalassemia, specifically for detecting carriers of β-thalassemia. However, the β-thalassemia trait is likely more prevalent in Indonesia. This study may overlook particular carriers of β-thalassemia. Additionally, individuals may exhibit iron deficiency anaemia in conjunction with thalassemia. Figure 2 shows a proposed algorithm for determining the underlying cause of microcytosis in stunted children.

### 2.7. Data Analysis

Statistical analysis is carried out with SPSS version 20, including descriptive analysis, correlation, comparison, and chi-square to examine the relationship between one dependent and more independent variables.

## 3. Results

A total of two hundred and ten stunted children under two years of age participated in this study. Based on Table 2, the children’s average age was 18 months, highlighting their early developmental stage, ranging from 1 to 24 months. The gender composition showed that 46.7% were males and 53.3% were females. The heights of both fathers and mothers showed that the majority had normal heights, with percentages of 77.6% and 71.9% respectively. Most mother participants also had a normal BMI (86.2%), and maternal education was predominantly at the secondary level, with 30.5%, 34.8%, and 9% having junior, senior high, and higher education, respectively. Most of the mothers were married between 17 and 20 (49.5%), and the majority had a full-term pregnancy (91.9%). About 11.9% of mothers had a history of anaemia during pregnancy. The mothers’ occupations were dominated by housewives (71.4%), while the fathers mainly were farmers (39.5%) and labourers (33.8%). In terms of family income, the majority of participants had an income of less than 1 million (46.2%) or 1–5 million (52.4%). There was a family history of disease, particularly TB, in 4.3% of families. Regarding the history of toddlers, the majority received exclusive breast milk (75.2%) and complete immunisation (73.8%). Table 2 displays the comprehensive clinical, maternal, and socioeconomic characteristics of stunted children. This information provides valuable insights into the complex factors contributing to childhood stunting.

Table 3 presents the distribution of haematology analyser blood test results, haematology index, and iron profile in stunted children.

The characteristics of haematological test results and haematological index of stunted children are shown in Table 4.

Based on Table 3, the median haemoglobin (Hb) level of stunted children was 11.3 g/dL. As shown in Table 4, 83 (39.5%) stunted children had Hb < 11 g/dL, indicating anaemia. The median corpuscular volume (MCV) was 71.9 with 134 (63.8%) having values < 74 fL, while 196 (93.3%) had median corpuscular haemoglobin (MCH) < 27 pg. This suggests that most stunted children had microcytic erythrocytes, possibly due to iron deficiency anaemia or thalassemia. The median red cell distribution width coefficient of variation (RDW CV) was 14.9%. About 106 (50.5%) stunted children had RDW CV > 15%, showing red cell characteristics of anisocytosis, possibly due to iron deficiency anaemia or thalassemia. Table 3 shows that the median serum iron level was 39 µg/dL, and ferritin was about 26, suggesting normoferremia with hyperferritinemia, possibly due to chronic diseases anaemia or thalassemia. About 182 (86.7%) had a Shine and Lai index of <1530, while 48 (22.9%) had an MI of <13, possibly showing thalassemia. The difference between the MI and the Shine and Lai Index lies in sensitivity and specificity in diagnosing thalassemia. The MI shows a sensitivity of 98.7% and a specificity of 82.3%, while the Shine and Lai Index have values of 100% and 10% respectively [11]. Haematological examination results showed significant variations in blood components, offering insights into the condition of stunted children. The iron profile variations in serum iron and ferritin levels can provide information about the nutritional status and iron metabolism. Additionally, inflammatory markers from haematological examinations offer insights into certain diseases that clinicians need to monitor [24]. The distribution of possible diseases in stunted children based on the haematology test and the inflammatory marker approach is shown in Table 5.

The results in Table 5 showed that the majority of suspected diseases were thalassemia (38.1%), followed by Allergic Proctocolitis (31.9%) and TB (21.4%). The possibility of disease in stunted children varies by region, depending on the culture and habits of different communities. In general, thalassemia is a major problem in North Maluku, with the percentage of patients reaching 51.0%. The number of patients with suspected thalassemia is also significant in West Java (36.5%) and West Nusa Tenggara (31.6%).

**Figure 3:**
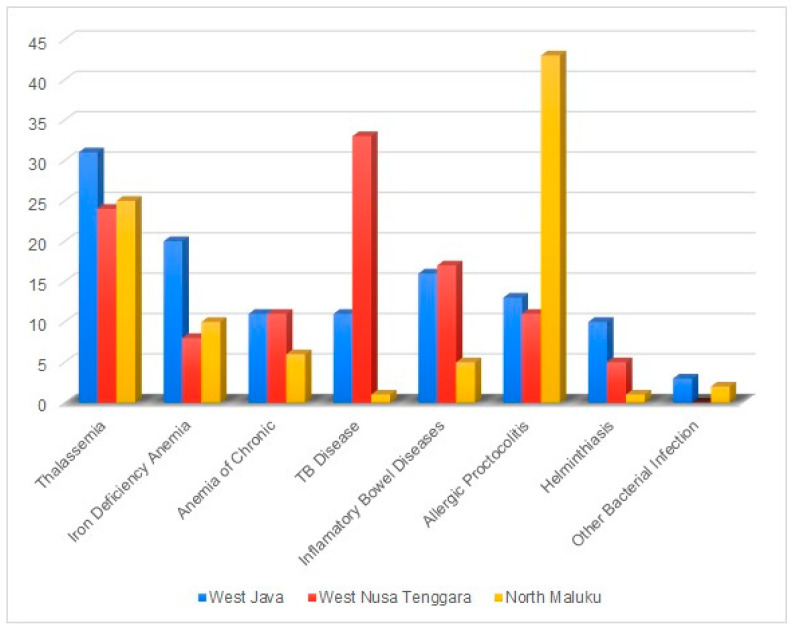
The proportion of possible diseases in stunted children.

Iron deficiency anaemia in West Java dominated with a percentage of 23.5%, while North Maluku also had a fairly high number (20.4%). Inflammatory bowel diseases suspected had an even distribution, but West Nusa Tenggara had the highest percentage (22.4%), while West Java had a figure of 18.8%. Allergic proctocolitis suspected showed significant differences, with North Maluku having a significantly higher rate (87.8%) compared to West Java (15.3%) and West Nusa Tenggara (14.5%). The proportion of possible diseases in stunted children is shown in Figure 3.

The correlation between haemoglobin with serum iron and ferritin is shown in Table 6.

**Figure 4:**
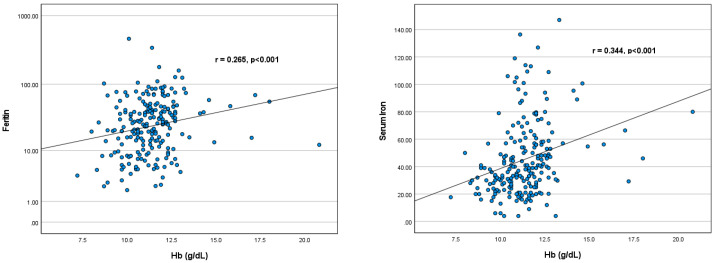
The Scatterplot of the Correlation between Haemoglobin and Iron Profile in Stunted Children.

Based on Table 6, the correlation coefficient (r) of serum iron is 0.265 with a *p*-value < 0.001. This indicates a significant positive correlation between haemoglobin levels and serum iron levels. The correlation coefficient of ferritin (r) is 0.344 with a *p*-value < 0.001. This also shows a significant positive correlation between haemoglobin levels and ferritin levels. Children with higher ferritin and iron serum levels tend to have higher haemoglobin levels. This shows that iron status plays a major role in determining haemoglobin levels in stunted children. The following Figure 4 shows the scatterplot of the correlation between Haemoglobin and iron profile in stunted children.

Table 7 compares anaemia profiles, inflammatory markers, and iron profiles by region in stunted children.

Based on a comparative analysis between haemoglobin, haematological parameters, and iron profiles in stunted children from three regions—West Java, West Nusa Tenggara, and North Maluku—several significant differences were found. There is a significant difference in haemoglobin levels between regions. Children in North Maluku have the highest haemoglobin levels with a median of 11.9 g/dL, followed by children in West Java with a median of 11.2 g/dL. Meanwhile, children in West Nusa Tenggara have the lowest haemoglobin levels with a median of 10.6 g/dL. These results indicate that the haemoglobin status of stunted children in North Maluku and West Java is better compared to children in West Nusa Tenggara, who have significantly lower haemoglobin status (*p* < 0.001).

**Figure 5:**
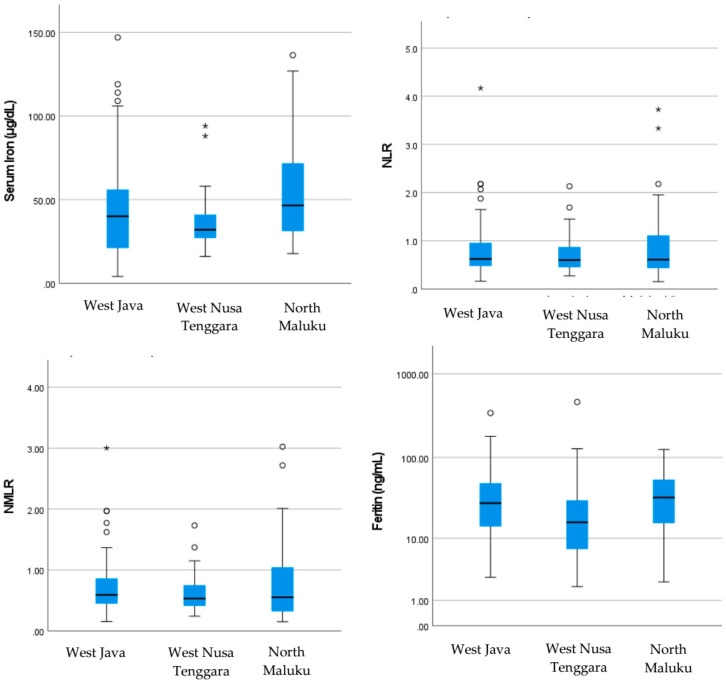
Boxplot comparison of haemoglobin and iron profile by region in stunted children. * means that a significant difference.

Based on inflammatory markers, only MPV had significant differences. Children in Lombok had the highest MPV with a median of 9.5 fL, compared to children in West Java and North Maluku who had lower MPV values of 8.1 fL and 8.2 fL, respectively. This may reflect differences in platelet activity or inflammatory response among children in West Nusa Tenggara compared to other areas (*p* < 0.001). There were significant differences in serum iron and ferritin levels. Children in North Maluku showed the highest serum iron levels with a median of 46.5 μg/dL, while children in West Nusa Tenggara had the lowest serum iron levels with a median of 32.0 μg/dL. The same was true for ferritin, where children in North Maluku had the highest ferritin reserves (median 32.7 ng/mL), while children in West Nusa Tenggara had the lowest ferritin reserves (median 16.0 ng/mL). This indicates that the iron status of children in West Nusa Tenggara was significantly lower compared to children in West Java and North Maluku (*p* = 0.001 for both). Boxplot comparison of haemoglobin and iron profiles by region in stunted children is shown in Figure 5.

The relationship between crucial characteristics and anaemia in stunted children is shown in Table 8.

Based on the analysis of the relationship between essential characteristics and the incidence of anaemia in stunted children, several factors were found to influence it significantly. Geographical location was shown to significantly affect the incidence of anaemia in stunted children (*p* = 0.002). Children in West Nusa Tenggara had the highest prevalence of anaemia (57.1%), while children in West Java had a prevalence of anaemia of 41.2%. In contrast, North Maluku’s children had the lowest anaemia prevalence (26.3%). Maternal and father’s occupations show significant relationships with the incidence of anaemia (*p* = 0.005 and *p* = 0.043, respectively).

## 4. Discussion

In the WHO conceptual framework, stunting is caused by the interaction of various factors, such as inadequate nutritional intake and increased nutritional needs. Insufficient intake can be caused by socioeconomic factors (poverty), low education and knowledge regarding feeding practices for infants and toddlers (sufficient breast milk), adequacy of animal protein in complementary foods for breast milk, neglect, cultural influences, and availability of local food ingredients. Factors causing an increase in demand include chronic diseases requiring food for special medical purposes. These include congenital heart disease, poor personal and environmental hygiene (chronic diarrhoea), as well as diseases that can be prevented by immunisation, such as tuberculosis, diphtheria, pertussis, and measles [4].

Children who are stunted usually co-exist with being underweight and wasting tend to have higher risks of illness and death. A study analysing data from 53,767 children in Africa, Asia, and Latin America found that the mortality rate was over three times higher in children who were stunted and underweight compared to well-nourished children [HR 3.4 (95%CI 2.6–4.3)]. For children with multiple anthropometric failures (stunted, wasted, and underweight), the mortality rate increased by more than twelve times. Stunted children often suffer from stunting syndrome, characterised by impaired growth, increased illness and death rates, and reduced physical, cognitive, and economic capabilities [6].

Based on Table 5, in the three regions, a significant number of suspected diseases in stunted children were identified as thalassemia (38.1%), possibly influenced by Indonesia’s location along the ‘Thalassemia Belt,’ making it a hotspot for hemoglobinopathies. β-thalassemia is carried by approximately 3.0–10.0% of the population, while α-thalassemia is carried by 2.6–11.0%. Around 2500 babies are estimated to be born with β-thalassemia major (β-TM) annually [25]. This highlights the importance of raising awareness and supporting affected individuals and families. In thalassemia patients, endocrine disorders occur, leading to stunting. Frequent and continuous transfusions can result in an unhealthy buildup of iron in the liver and endocrine glands, potentially leading to the development of endocrine disorders. Transfusion-dependent thalassemia (TDT) significantly impacts a child’s growth, potentially causing stunting, an increased risk of vitamin D deficiency, and reduced insulin-like growth factor-1 (IGF-1) levels. It is important to note that low IGF-1 levels are linked to stunting in children who undergo frequent transfusions [26].

Allergic proctocolitis (AP) is a non-IgE-mediated food allergy caused by immature immune systems, compromised intestinal permeability, genetic predisposition, and sensitivity to food antigens [27]. The study on paediatric patients with multiple food allergies found that abnormal production of TGF-β in the small intestine is a critical immunological issue [28]. Reduced expression of TGF-β1 in conjunction with a lack of innate immune response to gut commensal microbial flora may contribute to altered development of oral tolerance to foods [29]. Meanwhile, tumour necrosis factor-alpha (TNF-α) plays a crucial role in the development of IBD by affecting the tight junctions between epithelial cells, leading to changes in the intestinal barrier capacity. Therefore, tumour necrosis factor-alpha (TNF-α) appears to be involved in the pathogenesis of AP through the alteration of the intestinal epithelial barrier capacity. Heightened levels of TNF-α stimulate the production and activation of other pro-inflammatory cytokines and factors, fuelling intestinal inflammation. Understanding and addressing TNF-α is essential in managing these conditions [30].

As shown in Table 5, tuberculosis prevalence in stunted children was 21.4%, with the highest occurring in West Nusa Tenggara (43.4%). Tuberculosis and stunting are considered significant health problems in Indonesia. Nutritional status is a crucial factor in the occurrence of infectious diseases, including tuberculosis. The body can effectively fight infection well when provided with adequate amounts of nutritious food. In general, early childhood nutritional status plays a significant role in determining the ability to fight tuberculosis bacteria. Children with good nutrition can prevent the spread of disease in the lungs. However, those with malnutrition can be susceptible to lung disease with large cavities at an early age. In populations that have a large number of positive smear tuberculosis cases, many children will become ill with the disease. The risk of becoming ill with TB is highest among children under the age of three years [31]. A study across 22 countries found that 26% of tuberculosis cases are linked to malnutrition, highlighting the importance of addressing malnutrition to protect children from infections and tuberculosis. In Indonesia, a study found that stunted children had a much higher tuberculosis prevalence of 38.1% compared to other groups [19].

Based on the findings of Table 6, it can be concluded that iron is crucial for stunted children in preventing anaemia. Generally, anaemia in stunted children is caused by iron deficiency [8], and stunted children need to take iron tablets or consume iron-rich foods. Based on Table 7 as a whole, these results reveal clear differences in haemoglobin status and iron profiles in stunted children in the three regions. Children in North Maluku and West Java showed iron status that correlated with better nutritional status compared to children in West Nusa Tenggara, who appeared to experience lower iron status, potentially indicating greater health problems in those areas. These regional outcomes may reflect differences in access to nutrition, health care, or environmental factors.

Based on the results of Table 8, it can be concluded that geographical location was shown to have a significant effect on the incidence of anaemia in stunted children (*p* = 0.002). These regional differences may reflect differences in access to nutrition, health care, or environmental factors. Maternal occupation is one of the factors that shows a substantial relationship with the incidence of anaemia (*p* = 0.005). Children of mothers who worked as housewives had a higher prevalence of anaemia (45.7%). This shows that what plays a role in stunting in children is not the mother’s work but maybe parenting patterns in providing nutrition to children. Even though the majority of mothers with stunted children are homemakers, if the parenting style of delivering food is not good, it will affect the child’s health. Parenting and patterns of feeding are related to the incidence of stunting. Good maternal parenting can prevent children from experiencing stunting [32]. Further research needs to be conducted. Father’s occupation also shows a significant relationship with anaemia (*p* = 0.043), with children of fathers who work as private employees having the highest prevalence of anaemia (88.9%). Father’s occupation also shows a significant relationship with anaemia (*p* = 0.043), with children of fathers who work as private employees having the highest prevalence of anaemia (88.9%). The father’s work is related to the family’s income. This is also associated with the ability to provide nutritious nutrition to children.

This study has four significant limitations: (1) the gold standard examination was not conducted to confirm the screening results using haematology tools and inflammatory markers due to cost constraints. (2) Lack of anamnesis and physical examination by a clinician. (3) Small sample size due to limited timeframe. (4) There is no nutritional surveillance. (5) This research is descriptive and does not identify causal relationships. Therefore, the results do not show a direct causal relationship.

## 5. Conclusions

The findings suggest that stunted children under two exhibit a variety of anaemia profiles and potential comorbid conditions, including suspected thalassemia, iron deficiency, anaemia of chronic diseases, and inflammatory conditions. These results highlight the importance of conducting comprehensive anaemia and inflammation marker assessments in stunted children to understand the health complexities they may face better. It is recommended that these assessments be integrated into routine evaluations at the primary healthcare level to support more tailored interventions. However, further investigation is needed to confirm the underlying causes of stunting.

## Figures and Tables

**Table 1:** The Differential diagnosis of anaemia microcytosis.

Test	Suggested Diagnosis			
	Iron Deficiency Anaemia	Thalassemia	Anaemia of Chronic Disease	Sideroblastic Anaemia
Serum ferritin level	Decreased	Increased	Normal to increased	Normal to increased
Red blood cell distribution width (RDW)	Increased	Normal to increased	Normal	Increased
Serum iron level	Decreased	Normal to increased	Normal to decreased	Normal to increased
Total iron-binding capacity	Increased	Normal	Slightly decreased	Normal
Transferrin saturation	Decreased	Normal to increased	Normal to slightly increased	Normal to increased

Adapted with permission from Ref. [23].

**Table 2:** The clinical, maternal, and socioeconomic characteristics of stunted children.

Variable	n = 210
Stunted children’s age (Months)	
Median (IQR)	18 (13–22)
Min–Max	1–24
Gender, n (%)	
Male	98 (46.7)
Female	112 (53.3)
Father’s height, n (%)	
Short	47 (22.4)
Normal	163 (77.6)
Mother’s height, n (%)	
Short	59 (28.1)
Normal	151 (71.9)
Mother’s Body Mass Index, n (%)	
Thin	16 (7.6)
Normal	181 (86.2)
Overweight	13 (6.2)
Mother’s Education, n (%)	
No School	2 (1)
Elementary School	52 (24.8)
Junior High School	64 (30.5)
Senior High School	73 (34.8)
College	19 (9)
Maternal age at marriage, n (%)	
<17 years old	28 (13.3)
17–20 years old	104 (49.5)
21–30 years old	78 (37.1)
Gestational Age at Delivery, n (%)	
Preterm	16 (7.6)
Full-term	193 (91.9)
Post-term	1 (0.5)
Anaemia in mothers during pregnancy, n (%)	
Anaemia	25 (11.9)
No Anaemia	185 (88.1)
Mother’s occupation, n (%)	
Government employees	1 (0.5)
Labourer	4 (1.9)
Honorary employee	1 (0.5)
Housewife	150 (71.4)
Farmer	40 (19)
Entrepreneur	14 (6.6)
Father’s occupation, n (%)	
Government employees	1 (0.5)
Labourer	71 (33.8)
Fisherman	3 (1.4)
Merchant	1 (0.5)
Honorary employee	1 (0.5)
Farmer	83 (39.5)
Security	1 (0.5)
Not Working	1 (0.5)
Indonesian workers overseas	3 (1.4)
Entrepreneur	45 (21.4)
Family income each month, n (%)	
<1 million	97 (46.2)
1–5 million	110 (52.4)
5–10 million	3 (1.4)
History of disease in the family, n (%)	
History of TB diseases	9 (4.3)
TB active	10 (4.8)
HIV	-
Hepatitis	-
History in stunted children, n (%)	
Exclusive breastfeeding	
Yes	158 (75,2)
No	52 (24.8)
Providing Supplementary Food	
<6 Months	41 (19.5)
≥6 Months	169 (80.5)
Complete immunisation	155 (73.8)
History of TB diseases	20 (9.5)
History of HIV diseases	-
History of Hepatitis diseases	-

**Table 3:** Distribution of haematology analyser blood test results, haematology index, and iron profile in stunted children.

	Median (IQR)	Min–Maks
Haematology Analyser		
Hb (g/dL)	11.3 (10.4–12.0)	7.2–20.8
Hct (%)	4.9 (4.5–5.2)	3.5–8.8
RBC (10^6^/uL)	4.85 (4.545–5.1625)	3.51–8.78
MCV (fL)	71.9 (67.1–75.2)	46.5–90.0
MCH (pg)	23.7 (21.6–25.0)	14.2–33
MCHC (g/dL)	32.5 (31.4–34.0)	25.2–36
WBC (10^3^/uL)	10.4 (8.7–12.8)	4.6–24.7
PLT (10^3^/uL)	372 (282–435)	75–825
Eosinophils	3.6 (2–6)	0–25
Neutrophils	34.6 (27.0–44.0)	11.6–75
Lymphocytes	54.4 (44.3–61.0)	18.0–81.0
Monocytes	4.8 (2.7–7)	0.4–40.4
NLR	0.6 (0.5–1)	0.2–4.2
PLR	67.5 (45.2–91.5)	8.6–215.1
MLR	0.09 (0.05–0.15)	0.01–1.13
NMLR	0.6 (0.4–0.9)	0.2–3.0
MPV (fL)	8.45 (7.88–9.30)	6.6–12.1
RDW-CV (%)	14.9 (13.9–16.6)	11.3–23.7
RDW-SD (fL)	43.3 (40.7–45.6)	33.9–435
Haematology Index		
Mentzer	14.7 (13.2–16.1)	7.3–25.6
Shine and Lal	1210 (958–1413)	307–2673
Iron Profile		
Serum Iron	39 (27–57)	4–147
Ferritin	26.0 (12.0–45.8)	1.9–461.1

**Table 4:** Characteristics of haematological test results, and haematological index of stunted children.

Variable	n = 210
Haematology Analyser, n (%)	
Hb (g/dL)	
<11	83 (39.5)
≥11	127 (60.5)
RBC (10^6^/uL)	
<5.0	71 (33.8)
≥5.0	139 (66.2)
MCV (fL)	
<74	134 (63.8)
≥74	76 (36.2)
MCH (pg)	
<27	196 (93.3)
≥27	14 (6.7)
NLR	
>2.04	8 (3.8)
≤2.04	202 (96.2)
PLR	
>103	17 (20)
≤103	68 (65)
MLR	
>0.44	0 (0)
≤0.44	86(100)
NMLR	
>1.19	26 (12.4)
≤1.19	184 (87.6)
MPV (fL)	
>9	62 (29.5)
≤9	148 (70.5)
RDW-CV (%)	
<15	106 (50.5)
≥15	104 (49.5)
Haematology Index, n (%)	
Mentzer	
<13	48 (22.9)
≥13	162 (77.1)
Shine and Lal	
<1530	182 (86.7)
≥1530	28 (13.3)

**Table 5:** The Distribution of Possible Diseases in Stunted Children Based on the Haematology Test and Inflammatory Marker Approach.

Suspected Illness	All Subjects n (%)	West Java n (%)	West Nusa Tenggara n (%)	North Maluku n (%)
Thalassemia	80 (38.1)	31 (36.5)	24 (31.6)	25 (51.0)
Iron Deficiency Anaemia	38 (18.1)	20 (23.5)	8 (10.5)	10 (20.4)
Anaemia of Chronic Diseases	28 (13.3)	11 (12.9)	11 (14.5)	6 (12.2)
TB Diseases	45 (21.4)	11 (12.9)	33 (43.4)	1 (2.0)
IBD	38 (18.1)	16 (18.8)	17 (22.4)	5 (10.2)
Allergic Proctocolitis	67 (31.9)	13 (15.3)	11 (14.5)	43 (87.8)
Helminthiasis	16 (7.6)	10 (11.8)	5 (6.6)	1 (2.0)
Other Bacterial Infection	5 (2.4)	3 (3.5)	0 (0.0)	2 (4.1)
Total Subjects	210	85	76	49

**Table 6:** The correlation between haemoglobin with serum iron and ferritin.

Variable	Hb (g/dL)	
	r-Coefficient	*p*-Value
Serum Iron (μg/dL)	0.265	<0.001 *
Ferritin (ng/mL)	0.344	<0.001 *

Analysis with Spearman’s rho, * significant.

**Table 7:** The comparison of anaemia profiles, inflammatory markers, and iron profiles by region in stunted children.

Variable	West Java (n = 85)	West Nusa Tenggara (n = 76)	North Maluku (n = 49)	Nilai *p*
	Median (IQR)	Median (IQR)	Median (IQR)	
Haematology Analyser				
Haemoglobin (g/dL)	11.2 (10.6–11.9)	10.6 (9.9–11.6)	11.9 (10.7–12.6)	<0.001 *
NLR	0.62 (0.48–0.97)	0.60 (0.45–0.88)	0.61 (0.43–1.12)	0.727
NMLR	0.59 (0.44–0.86)	0.53 (0.41–0.76)	0.55 (0.32–1.05)	0.407
MPV (fL)	8.1 (7.7–8.7)	9.5 (9.1–10.5)	8.2 (7.6–8.7)	<0.001 *
Iron Profile				
Serum Iron (μg/dL)	40.0 (20.5–56.5)	32.0 (26.5–41.0)	46.5 (30.9–71.8)	0.001 *
Ferritin (ng/mL)	27.8 (14.2–48.7)	16.0 (6.7–30.2)	32.7 (15.5–54.6)	0.001 *

Analysis using the Kruskall Wallis test, * significant.

**Table 8:** The relationship between essential characteristics and the occurrence of anaemia in Stunted children.

Variable	Total	Group		*p*-Value
		Anaemia (Hb < 11 g/dL) n = 83	Normal (Hb ≥ 11 g/dL) n = 100	
Site, n (%)				0.002 *
West Java	85	35 (41.2)	50 (58.8)	
West Nusa Tenggara	49	28 (57.1)	21 (42.9)	
North Maluku	76	20 (26.3)	56 (73.7)	
Gender, n (%)				0.355
Male	98	42 (42.9)	56 (57.1)	
Female	112	41 (36.6)	71 (63.4)	
Father’s height, n (%)				0.594
Short	47	17 (36.2)	30 (63.8)	
Normal	163	66 (40.5)	97 (59.5)	
Mother’s height, n (%)				0.248
Short	59	27 (45.8)	32 (54.2)	
Normal	151	56 (37.1)	95 (62.9)	
Mother’s Body Mass Index, n (%)				0.074
Thin	16	9 (56.3)	7 (43.8)	
Normal	181	66 (36.5)	115 (63.5)	
Overweight	13	8 (61.5)	5 (38.5)	
Mother’s Education, n (%)				0.304
No School	2	2 (100)	0 (0)	
Elementary School	52	21 (40.4)	31 (59.6)	
Junior High School	64	24 (37.5)	40 (62.5)	
Senior High School	73	31 (42.5)	42 (57.5)	
College	19	5 (26.3)	14 (73.7)	
Maternal age at marriage, n (%)				0.885
<17 years old	28	10 (35.7)	18 (64.3)	
17–20 years old	104	41 (39.4)	63 (60.6)	
21–30 years old	78	32 (41.0)	46 (59.0)	
Gestational Age at Delivery, n (%)				0.707
Preterm	16	6 (37.5)	10 (62.5)	
Full-term	193	77 (39.9)	116 (60.1)	
Post-term	1	0 (0)	1 (100)	
Anaemia in mothers during pregnancy, n (%)				0.091
Anaemia	25	6 (24.0)	19 (76.0)	
No Anaemia	185	77 (41.6)	108 (58.4)	
Mother’s occupation, n (%)				0.005 *
Housewife	151	69 (45.7)	82 (54.3)	
Farmer	40	7 (17.5)	33 (82.5)	
Employee/labourer	19	7 (36.8)	12 (63.2)	
Father’s occupation, n (%)				0.043 *
Not working	4	1 (25.0)	3 (75.0)	
Honorary Employee	9	8 (88.9)	1 (11.1)	
Farmer/Fisherman	86	32 (37.2)	54 (62.8)	
Entrepreneur	40	15 (37.5)	25 (62.5)	
Labourer	71	27 (38.0)	44 (62.0)	
Family income each month, n (%)				0.603
<1 million	97	37 (38.1)	60 (61.9)	
1–5 million	110	44 (40)	66 (60)	
5–10 million	3	2 (66.7)	1 (33.3)	
History of disease in the family, n (%)				
History of TB diseases				0.278
Yes	9	2 (22.2)	7 (77.8)	
No	201	81 (40.3)	120 (59.7)	
TB active				0.528
Yes	10	3 (30)	7 (70)	
No	200	80 (40)	120 (60)	
History in stunted children, n (%)				
Exclusive breastfeeding				0.884
Yes	158	62 (39.2)	96 (60.8)	
No	52	21 (40.4)	31 (59.6)	
Providing Supplementary Food				0.134
<6 Months	41	12 (29.3)	29 (70.7)	
≥6 Months	169	71 (42)	98 (58)	
Complete immunisation				0.685
Yes	155	60 (38.7)	95 (61.3)	
No	55	23 (41.8)	32 (58.2)	
TB History				0.360
Yes	20	6 (30)	14 (70)	
No	190	77 (40.5)	113 (59.5)	

Analysis using Chi-square test, * significant *p* < 0.05.

## Data Availability

Patient confidentiality is maintained by storing data in the researcher’s Google Drive. Data can only be accessed and shared with the researcher’s permission. The link for this study data is https://drive.google.com/drive/folders/152O_J1-uRMmWb6SmsFkgplR5fC_iAcOn?usp=drive_link (accessed on 15 February 2024).

## Supplementary Materials

The following supporting information can be downloaded at: https://www.mdpi.com/article/10.3390/children11111315/s1, File S1: Data of Participant.

## Abbreviations

GHI	Global Hunger Index
IBD	Inflammatory Bowel Diseases
NLR	Neutrophil/Lymphocyte Ratio
HAZ	Height-for-age
Hb	Haemoglobin
Hct	Haematocrit
MCHC	Mean Corpuscular Haemoglobin Concentration
MCV	Mean Corpuscular Volume
MLR	Monocyte/Lymphocyte Ratio
MPV	Mean Platelet Volume
NMLR	Neutrophil/Lymphocyte + Monocyte Ratio
WBC	White Blood Cell
WHO	World Health Organization
PLR	Platelet/Lymphocyte Ratio
RBC	Red Blood Cell
RDW CV	Red Cell Distribution Wide Coefficient Variation
TB	Tuberculosis

## References

[1] Estimates J.C.M., WHO (2023). Levels and Trends in Child Malnutrition.

[2] von Grebmer K., Bernstein J., Wiemers M., Reiner L., Bachmeier M., Hanano A. (2023). Global Hunger Index the Power of Youth in Shaping Food Systems.

[3] Kemenkes (2022). Pocket Book: Indonesian Nutritional Status Survey (SSGI) 2022.

[4] WHO (2014). Global Nutrition Targets 2025: Stunting Policy Brief.

[5] Addo O.Y., Stein A.D., Fall C.H., Gigante D.P., Guntupalli A.M., Horta B.L., Kuzawa C.W., Lee N., Norris S.A., Prabhakaran P. (2013). Maternal height and child growth patterns. J. Pediatr..

[6] Prendergast A.J., Humphrey J.H. (2014). The stunting syndrome in developing countries. Paediatr. Int. Child. Health.

[7] Abdullah A. (2015). The Double Burden of Undernutrition and Overnutrition in Developing Countries: An Update. Curr. Obes. Rep..

[8] WHO Haemoglobin Concentrations for the Diagnosis of Anaemia and Assessment of Severity 2011. https://iris.who.int/bitstream/handle/10665/85839/WHO_NMH_NHD_MNM_11.1_eng.pdf?sequence=22.

[9] TNP2K (2017). 100 Priority Districts/Cities for Stunting Intervention.

[10] Maskoen A.M., Reniarti L., Sahiratmadja E., Sisca J., Effendi S.H. (2019). Shine & Lal index as a predictor for early detection of beta-thalassemia carriers in a limited resource area in Bandung, Indonesia. BMC Med. Genet..

[11] Vehapoglu A., Ozgurhan G., Demir A.D., Uzuner S., Nursoy M.A., Turkmen S., Kacan A. (2014). Haematological indices for differential diagnosis of Beta thalassemia trait and iron deficiency anaemia. Anemia.

[12] Gysi S., Doulberis M., Legeret C., Kohler H. (2022). The Role of the Pediatric Yorkhill Malnutrition Score (PYMS), Neutrophil-to-Lymphocyte and Platelet-to-Lymphocyte Ratios in Malnutrition Prediction of Hospitalized Children. Children.

[13] Domnicu A.E., Boia E.R., Mogoi M., Manea A.M., Marcovici T.M., Marginean O., Boia M. (2023). The Neutrophil-to-Lymphocyte Ratio (NLR) Can Predict Sepsis’s Presence and Severity in Malnourished Infants Single Center Experience. Children.

[14] Zahmatkesh A., Sohouli M.H., Hosseini S.M.E., Rohani P. (2023). The role of platelet-to-lymphocyte ratio and neutrophil-to-lymphocyte ratio in the diagnosis and severity of inflammatory bowel disease in children. Eur. J. Pediatr..

[15] Nacaroglu H.T., Bahceci Erdem S., Durgun E., Karaman S., Baris Erdur C., Unsal Karkmer C.S., Can D. (2018). Markers of inflammation and tolerance development in allergic proctocolitis. Arch. Argent. Pediatr..

[16] Sabin F.R., Sugiyama S., Kindwall J.A., Cunningham R.S. (1925). The role of the monocyte in tuberculosis. B Johns. Hopkins Hosp..

[17] Kissling M., Fritschi N., Baumann P., Buettcher M., Bonhoeffer J., Naranbhai V., Ritz N. (2023). Monocyte, Lymphocyte and Neutrophil Ratios—Easy-to-Use Biomarkers for the Diagnosis of Pediatric Tuberculosis. Pediatr. Infect. Dis. J..

[18] Cursi L., Lancella L., Mariani F., Martino L., Leccese B., Di Giuseppe M., Venuti F., Cristina R., Gentile L., Sali M. (2023). Monocyte-to-lymphocyte, neutrophil-to-lymphocyte, and neutrophil-to-monocyte plus lymphocyte ratios in children with active tuberculosis: A multicentre study. Acta Paediatr..

[19] Ministry of Health of The Republic of Indonesia (2022). Decree of The Ministry of Health of the Republic Indonesia.

[20] WHO (2000). The Asia-Pacific Perspective: Redefining Obesity and Its Treatment.

[21] Gaston R.T., Habyarimana F., Ramroop S. (2022). Joint modelling of anaemia and stunting in children less than five years of age in Lesotho: A cross-sectional case study. BMC Public Health.

[22] Mohammed S.H., Larijani B., Esmaillzadeh A. (2019). Concurrent anaemia and stunting in young children: Prevalence, dietary and non-dietary associated factors. Nutr. J..

[23] Van Vranken M. (2010). Evaluation of microcytosis. Am. Fam. Physician.

[24] Braat S., Pasricha S.R. (2021). Finding ferritin in the plateaus and valleys of iron deficiency. Lancet Haematol..

[25] Wahidiyat P.A., Sari T.T., Rahmartani L.D., Iskandar S.D., Pratanata A.M., Yapiy I., Setianingsih I., Atmakusuma T.D., Lubis A.M. (2022). Thalassemia in Indonesia. Hemoglobin.

[26] Pratiwi I.G.A.P.E., Irawan R., Ugrasena I.D.G., Faizi M. (2022). Vitamin D, insulin-like growth factor-1, and stunting in children with transfusion-dependent thalassemia. Paediatr. Indones..

[27] Barni S., Mori F., Giovannini M., Liotti L., Mastrorilli C., Pecoraro L., Saretta F., Castagnoli R., Arasi S., Caminiti L. (2023). Allergic Proctocolitis: Literature Review and Proposal of a Diagnostic-Therapeutic Algorithm. Life.

[28] Chehade M., Mayer L. (2005). Oral tolerance and its relation to food hypersensitivities. J. Allergy Clin. Immunol..

[29] Perez-Machado M.A., Ashwood P., Thomson M.A., Latcham F., Sim R., Walker-Smith J.A., Murch S.H. (2003). Reduced transforming growth factor-beta1-producing T cells in the duodenal mucosa of children with food allergy. Eur. J. Immunol..

[30] Ma T.Y., Iwamoto G.K., Hoa N.T., Akotia V., Pedram A., Boivin M.A., Said H.M. (2004). TNF-alpha-induced increase in intestinal epithelial tight junction permeability requires NF-kappa B activation. Am. J. Physiol. Gastrointest. Liver Physiol..

[31] Haerana B.T., Prihartono N.A., Riono P., Djuwita R., Syarif S., Hadi E.N., Kaswandani N. (2021). Prevalence of tuberculosis infection and its relationship to stunting in children (under five years) household contact with new tuberculosis cases. Indian J. Tuberc..

[32] Pradana Putri A., Rong J.R. (2021). Parenting functioning in stunting management: A concept analysis. J. Public Health Res..

