# Supplementary material for: Anaemia Profile and Inflammation Markers in Stunted Children Under Two Years in Indonesia
Source: Children (Basel). 2024 Oct 29;11(11):1315. doi: 10.3390/children11111315 (PMC11592781; doi:10.3390/children11111315)
Supplement: Supplementary file 1 [file children-11-01315-s001.zip › children-3253267-supplementary.pdf]

No	Site	Sample Code	Code	Name
1	Jabar -PL	S001	Jabar	SUCI RAMADANI
2	Jabar -PL	S002	Jabar	IQLIMA UMMUSALAMA
3	Jabar -PL	S003	Jabar	MUHAMMAD ADTYA ASKARI
4	Jabar -PL	S004	Jabar	HALIMAH ULFATULZAHRA
5	Jabar -PL	S005	Jabar	TAZKIA
6	Jabar -PL	S006	Jabar	SUSI NURHAPISAH
7	Jabar -PL	S007	Jabar	RIFAN
8	Jabar -PL	S008	Jabar	MUHAMAD RAMDAN
9	Jabar -PL	S009	Jabar	DEDE LARAS
10	Jabar -PL	S010	Jabar	M.FAISAL
11	Jabar -PL	S011	Jabar	DIANA PUTRI
12	Jabar -PL	S012	Jabar	M.RIFALDI
13	Jabar -PL	S013	Jabar	HAIDAR
14	Jabar -PL	S014	Jabar	FRADIVA RAKA
15	Jabar -PL	S015	Jabar	M.EZHAR RAMADAN
16	Jabar -PL	S016	Jabar	HAURA ALMAYHYRA
17	Jabar -PL	S017	Jabar	NAURA ARSYILA
18	Jabar -PL	S018	Jabar	NURJAMAN
19	Jabar -PL	S019	Jabar	NAZRIL
20	Jabar -PL	S020	Jabar	RINKA NADIRA
21	Jabar -PL	S021	Jabar	HANUM NURFADILAH
22	Jabar -PL	S022	Jabar	ANISA
23	Jabar -PL	S023	Jabar	BUNGA LESTARI
24	Jabar -PL	S024	Jabar	CINDY
25	Jabar -PL	S025	Jabar	SAZIA NADIRA AZAHRA
26	Jabar -PL	S026	Jabar	WILDAN ZULFIKAR
27	Jabar -PL	S027	Jabar	AISYAH
28	Jabar -PL	S028	Jabar	RAYNA
29	Jabar -PL	S029	Jabar	FAISAL
30	Jabar -PL	S030	Jabar	ANAYA PUTRI
31	Jabar -PL	S031	Jabar	RAYA RAMBU RABANI
32	Jabar -PL	S032	Jabar	M.RIAN SAPUTRA
33	Jabar -PL	S033	Jabar	M.FAUJAN RAMADAN
34	Jabar -PL	S034	Jabar	NAIRA
35	Jabar -PL	S035	Jabar	M.ZULFIAN PRATAMA
36	Jabar -PL	S036	Jabar	BUKORI
37	Jabar -PL	S037	Jabar	M. ABDUL HADI
38	Jabar -PL	S038	Jabar	SYAKIRA RAHMAWATI
39	Jabar -PL	S039	Jabar	TANZIL AJIZ
40	Jabar -PL	S040	Jabar	NADIRA
41	Jabar -PL	S041	Jabar	ANISA
42	Jabar -PL	S042	Jabar	ANIA
43	Jabar -PL	S043	Jabar	RAPASHA NIZAM
44	Jabar -Cisaat	C001	Jabar	KABA MUHYI RASYID
45	Jabar -Cisaat	C002	Jabar	M ARSYAKA IBNU AZIS
46	Jabar -Cisaat	C003	Jabar	M DALVI
47	Jabar -Cisaat	C004	Jabar	YAHYA
48	Jabar -Cisaat	C005	Jabar	RIZHANI
49	Jabar -Cisaat	C006	Jabar	RAHMI RAMADHANI PUTRI

50	Jabar -Cisaat	C007	Jabar	RIZKYA MUTIA AZAHRA
51	Jabar -Cisaat	C008	Jabar	ARZAM
52	Jabar -Cisaat	C009	Jabar	KINTANI
53	Jabar -Cisaat	C010	Jabar	MEYSHA
54	Jabar -Cisaat	C011	Jabar	KYOMI ALKA FAHIMA
55	Jabar -Cisaat	C012	Jabar	RILIS APRIANI
56	Jabar -Cisaat	C013	Jabar	ZADIYYA
57	Jabar -Cisaat	C014	Jabar	TADIYYA
58	Jabar -Cisaat	C015	Jabar	KHANSA
59	Jabar -Cisaat	C016	Jabar	CUT ASSYURA PUTRI
60	Jabar -Cisaat	C017	Jabar	SILSI SILVIANA
61	Jabar -Cisaat	C018	Jabar	AMMAR YUSUF ABQORI
62	Jabar -Cisaat	C019	Jabar	MUHAMMAD FADEL ALFAYAD
63	Jabar -Cisaat	C020	Jabar	ARVI MAHARDIKA
64	Jabar -Cisaat	C021	Jabar	SAEPUL MUHAMMAD ABDULLAH
65	Jabar -Cisaat	C022	Jabar	ELEANDRA JENNAIRA KHAYYARA
66	Jabar -Cisaat	C023	Jabar	NADIA FATIMATUZ ZAHRA
67	Jabar -Cisaat	C024	Jabar	JAUZA
68	Jabar -Cisaat	C025	Jabar	MUHAMMAD MUMTAAZ M
69	Jabar -Cisaat	C026	Jabar	GHIFARI FALAH ALADNAN
70	Jabar -Cisaat	C027	Jabar	DWI HAIFA
71	Jabar -Cisaat	C028	Jabar	ADZKYA NAILA
72	Jabar -Cisaat	C029	Jabar	SRI MULYANI
73	Jabar -Cisaat	C030	Jabar	NAZWA MAULIDA
74	Jabar -Cisaat	C031	Jabar	NAZEERA SAFIYYA
75	Jabar -Cisaat	C032	Jabar	SITI INARA
76	Jabar -Cisaat	C033	Jabar	EGA ADITYA PERMANA
77	Jabar -Cisaat	C034	Jabar	M RAZKA MUMTAZ
78	Jabar -Cisaat	C035	Jabar	ALBIAN
79	Jabar -Cisaat	C036	Jabar	AZKILA
80	Jabar -Cisaat	C037	Jabar	BIRU EZAZ HENDRISAL
81	Jabar -Cisaat	C038	Jabar	TAZKIA
82	Jabar -Cisaat	C039	Jabar	SHABIRA HAIFA HUSNIYA
83	Jabar -Cisaat	C040	Jabar	TSABITUL AZMI
84	Jabar -Cisaat	C041	Jabar	MAHIRA
85	Jabar -Cisaat	C042	Jabar	NAZEEFA SAFIYAH
86	MU - Talagamori	T01	Malut	ALFANDI ISRA
87	MU - Talagamori	T02	Malut	MUH. FATIH
88	MU - Talagamori	T03	Malut	RAMADANI
89	MU - Talagamori	T04	Malut	FANI FARSAM
90	MU - Talagamori	T05	Malut	NURALAYKL
91	MU - Talagamori	T06	Malut	RISKIANA ALIFA
92	MU - Talagamori	T07	Malut	ELIZA JINAIRA
93	MU - Talagamori	T08	Malut	FAINA IRFANDI
94	MU - Talagamori	T09	Malut	NURSHAFANA
95	MU - Talagamori	T10	Malut	NURSAHNAYA
96	MU - Talagamori	T11	Malut	LESTARI
97	MU - Talagamori	T12	Malut	RAMLIA NURLILI
98	MU - Talagamori	T13	Malut	ALVIR HAMDAN
99	MU - Talagamori	T14	Malut	SUSANTI
100	MU - Talagamori	T15	Malut	MEIRA SYAMSUDIN
101	MU - Talagamori	T16	Malut	MUHAZZAM
102	MU - Talagamori	T17	Malut	SANDRA HUSAIN

103	MU - Talagamori	T18	Malut	M.ASKA
104	MU - Talagamori	T19	Malut	NAIMA.R
105	MU - Talagamori	T20	Malut	NAIZAR
106	MU - Pahaye	T22	Malut	M. FIKAR SUHARDI
107	MU - Pahaye	T23	Malut	AFRIJAL
108	MU - Pahaye	T24	Malut	RAFFASYA ALFARIQ
109	MU - Pahaye	T25	Malut	MUH. ALHIDAYAT. N
110	MU - Pahaye	T26	Malut	AUREL
111	MU - Pahaye	T27	Malut	HAJIKO NANDOHE
112	MU - Pahaye	T28	Malut	IRENE SAMA
113	MU - Pahaye	T29	Malut	M. RAFI
114	MU - Pahaye	T30	Malut	SITI HAJRA. M
115	MU - Pahaye	T31	Malut	NURKHALISA IDHAM
116	MU - Pahaye	T32	Malut	ASSURA SABILA
117	MU - Pahaye	T33	Malut	M. RAFIDAN
118	MU - Rum & Ome	T34	Malut	ASMIHANDA
119	MU - Rum & Ome	T35	Malut	ALSYATUL MUFIDAH
120	MU - Rum & Ome	T36	Malut	RABIATUL ADAWIA
121	MU - Rum & Ome	T37	Malut	HABIBI ALBY YUSRIL
122	MU - Rum & Ome	T38	Malut	FAHRIZA TALID
123	MU - Rum & Ome	T39	Malut	MAHER MANSUR
124	MU - Rum & Ome	T40	Malut	FIRHAN MUH
125	MU - Rum & Ome	T41	Malut	RISALDI
126	MU - Rum & Ome	T42	Malut	RUDIA ASIS
127	MU - Rum & Ome	T43	Malut	RAIDAN AJALA
128	MU - Rum & Ome	T44	Malut	SYIFA SAUKIYA
129	MU - Rum & Ome	T45	Malut	M. ALFARIZI ODENTE
130	MU - Rum & Ome	T46	Malut	LIRFAN MOHTAR
1	MU - Rum & Ome	T47	Malut	KHALIFA IDRIS
132	MU - Rum & Ome	T48	Malut	FARADILA ATMOJO
133	MU - Rum & Ome	T50	Malut	ASLAN HIDAYAT
134	MU - Tosa	T51	Malut	SAHRADEN WAHID
135	MU - Tosa	T52	Malut	ASRAN M. JABIR
136	MU - Tosa	T53	Malut	M. IRSYAD
137	MU - Tosa	T54	Malut	RAHMADINA RAHMAN
138	MU - Tosa	T55	Malut	DAFFA AZRIEL
139	MU - Tosa	T56	Malut	NAZRAH ALWAN
140	MU - Tosa	T57	Malut	LIA HAFIZAH
141	MU - Tosa	T58	Malut	SRIFANI YUNUS
142	MU - Tosa	T59	Malut	M. SEPYAN TODUHO
143	MU - Tosa	T60	Malut	M. JANUAREZA TODUHO
144	MU- Tomalow	T62	Malut	NADIRA
145	MU- Tomalow	T63	Malut	NADARA
146	MU- Tomalow	T64	Malut	M. SAIDUL ANAS
147	MU- Tomalow	T65	Malut	SHANUM SAMARA
148	MU- Tomalow	T67	Malut	NAIRA ADZRA
149	MU- Tomalow	T68	Malut	ZULAYKA MIQAILA
150	MU - Soa Sio	T69	Malut	AISWA NAILA TE TODUHO
151	MU - Soa Sio	T70	Malut	JASMITY NUR ANISA
152	MU - Soa Sio	T71	Malut	NAHDATUL AZAHRA IWAN
153	MU - Soa Sio	T72	Malut	MUHAMMAD ALZAHABI
154	MU - Soa Sio	T73	Malut	ARKAN ALFATIH
155	MU_Dokiri	T85	Malut	NABILA JAINUDDIN

156	MU_Dokiri	T88	Malut	ANISA HUSNI
157	MU_Dokiri	T93	Malut	DAFFA ALFARIZKY
158	MU-Topo	T97	Malut	NOVA ISMALIYANTI
159	MU-Topo	T104	Malut	PUTRA FARIDAN U
160	MU-Topo	T105	Malut	SULHAN SUKARDI
161	MU-Gurabunga	T106	Malut	NAURA AGUS SALIM
162	Lombok	2	Lombok	Dianda Fatia
163	Lombok	3	Lombok	Muhammad Faiz
164	Lombok	6	Lombok	Muhammad Akbar
165	Lombok	7	Lombok	Zafran Khairi
166	Lombok	8	Lombok	Ali Abdurrahman
167	Lombok	9	Lombok	Ilmi Zahira
168	Lombok	10	Lombok	Jesica Alfira
169	Lombok	11	Lombok	Muhammad Izah Algifari
170	Lombok	12	Lombok	Hadi Taufik
171	Lombok	13	Lombok	Albian
172	Lombok	14	Lombok	Khofifah
173	Lombok	15	Lombok	Sobri Al-Azmi
174	Lombok	16	Lombok	Muhammad Farel
175	Lombok	17	Lombok	Fazia
176	Lombok	18	Lombok	Roza Alfian Haris
177	Lombok	19	Lombok	Zafran Chairi
178	Lombok	20	Lombok	Ahmad Fahrul Rozi
179	Lombok	22	Lombok	Yesa Dania Palas
180	Lombok	23	Lombok	Lutfi Abi Manggala
181	Lombok	24	Lombok	Nurhayatul Ilmi
182	Lombok	25	Lombok	Muhammad Rizki Akbar
183	Lombok	26	Lombok	Ahtar Ramadhan Putra
184	Lombok	27	Lombok	Baiq Kiki Purnama
185	Lombok	28	Lombok	Halifa Zea Amanda
186	Lombok	29	Lombok	Muh El Bilal
187	Lombok	30	Lombok	Demas Zakar
188	Lombok	32	Lombok	Nizam Al-Fikri
189	Lombok	33	Lombok	Nilem Cahaya
190	Lombok	34	Lombok	Halil Rafa
191	Lombok	35	Lombok	Muhammad Ramdhani Akbar
192	Lombok	36	Lombok	Rafael Faizal Akbar
193	Lombok	37	Lombok	Laili Lidia
194	Lombok	38	Lombok	Zulkarnaen
195	Lombok	39	Lombok	Ahmad Syakir Alzi
196	Lombok	40	Lombok	Putri Elmanisa Rahma
197	Lombok	41	Lombok	Khairil Anzala
198	Lombok	43	Lombok	Kayla Niza Khodijah
199	Lombok	44	Lombok	Zakir
200	Lombok	45	Lombok	Fathin Ardiana
201	Lombok	46	Lombok	Aulia Tamim Mufazal
202	Lombok	47	Lombok	Herlin Zahra
203	Lombok	48	Lombok	Zahda Jauratun
204	Lombok	49	Lombok	Ahmad Fakih Prayoga
205	Lombok	50	Lombok	Ahmad Jaya Prana
206	Lombok	51	Lombok	Antoni Arga
207	Lombok	52	Lombok	Azril Sya'bani Rizki
208	Lombok	53	Lombok	Hawa Azkia Azhar

209	Lombok	54	Lombok	Supiatun M.
210	Lombok	55	Lombok	Fadhina Ayu Wardhani
